# Early Season Monitoring of Tarnished Plant Bug, *Lygus lineolaris*, in Wild Hosts Using Pheromone Traps

**DOI:** 10.3390/insects14100805

**Published:** 2023-10-07

**Authors:** Justin George, James P. Glover, Gadi V. P. Reddy, Chris Johnson, David R. Hall

**Affiliations:** 1 Southern Insect Management Research Unit, USDA-ARS, Stoneville, MS 38776, USA; james.glover@usda.gov (J.P.G.); gadi.reddy@usda.gov (G.V.P.R.); chris.johnson@usda.gov (C.J.); 2 Natural Resources Institute, University of Greenwich, Chatham Maritime, Kent ME4 4TB, UK; d.r.hall@greenwich.ac.uk

**Keywords:** tarnished plant bug, sex pheromones, wild hosts, weeds, cotton, monitoring, sampling, pheromone traps, visual cues

## Abstract

**Simple Summary:**

The tarnished plant bug, *Lygus lineolaris* (Hemiptera: Miridae), is a polyphagous pest and causes severe economic damage to cotton crops. Managing the weedy field edges is important in preventing early-season infestations of *L. lineolaris* in cotton to prevent damage to the squares and other fruiting structures. Scouting fields for *L. lineolaris* is time- and labor-intensive, and end-user variability associated with field sampling can lead to inaccuracies. Insect traps that combine visual cues and pheromones are more accurate, sustainable, and economically feasible in contrast to traditional insect detection methods. In this study, we investigated the application of red or white sticky cards baited with the female-produced sex pheromone to monitor the overwintering *L. lineolaris* populations in early spring. Field experiments demonstrated that the red sticky cards baited with a pheromone blend containing hexyl butyrate, (*E*)-2-hexenyl butyrate, and (*E*)-4-oxo-2-hexenal in a 4:10:7 ratio are highly effective in trapping *L. lineolaris* adults in early spring before the row crops are planted, and in monitoring their movement into a cotton crop.

**Abstract:**

The tarnished plant bug, *Lygus lineolaris* (Hemiptera: Miridae), has a wide host range of over 700 plant species, including 130 crops of economic importance. During early spring, managing the field edges with weeds and other wild hosts is important in preventing early-season infestations of *L. lineolaris* in cotton to prevent damage to the squares and other fruiting structures. Scouting fields for *L. lineolaris* is time- and labor-intensive, and end-user variability associated with field sampling can lead to inaccuracies. Insect traps that combine visual cues and pheromones are more accurate, sustainable, and economically feasible in contrast to traditional insect detection methods. In this study, we investigated the application of red or white sticky cards baited with the female-produced sex pheromone to monitor overwintering *L. lineolaris* populations in early spring. Field experiments demonstrated that the red sticky cards baited with a pheromone blend containing hexyl butyrate, (*E*)-2-hexenyl butyrate, and (*E*)-4-oxo-2-hexenal in 4:10:7 ratio are highly effective in trapping *L. lineolaris* adults in early spring before the row crops are planted, and in monitoring their movement into a cotton crop. The monitoring of *L. lineolaris* should help growers to make judicious decisions on insecticide applications to control early pest infestations, thereby reducing economic damage to cotton.

## 1. Introduction

Cotton, *Gossypium hirsutum* L., is one of the world’s most widely grown agricultural crop with a production of 118.5 million bales from 32.8 million hectares in 2022–2023 [1]. The United States (US) is ranked the third largest producer of cotton, with a production of 17.6 million bales, behind India (27 mi) and China (27 mi) [2]. Several insect pests cause economic damage to cotton across the developmental stages of the crop. The tarnished plant bug, *Lygus lineolaris* (Palisot de Beauvois) (Hemiptera: Miridae), is considered one of the most economically important pests of cotton as their feeding can significantly reduce the crop yield due to the abscission of squares, boll malformation, and deformities of plant terminus [3]. Eradication programs of Boll weevil (*Anthonomus grandis* Boheman) (Coleoptera: Curculionidae), coupled with the area-wide use of transgenic *Bacillus thuringiensis* (Bt) against caterpillar pests, has caused *L. lineolaris* to emerge as a key pest in cotton-growing regions of the US. Management of this pest is heavily dependent on insecticides and requires multiple applications to minimize economic loss [4]. The pest status of this insect pest has increased significantly in the past two decades, resulting in a multi-fold surge in the number of insecticide applications [5,6].

*Lygus lineolaris* is a polyphagous pest and is reported to feed on more than 700 plant species in 55 families in North America [7]. The spatial distribution of economically important crops and weedy hosts in an agroecosystem significantly affects this pest’s movement and population dynamics early in the season [8]. In early spring, weed hosts are a major refuge for *L. lineolaris* in the absence of agricultural crops. Once the weedy hosts senesce or are destroyed, the insects migrate into adjacent crop fields and cause damage to the cotton squares [7,9,10]. Decisions on insecticide application for managing *L. lineolaris* are based primarily on economic thresholds developed by scouting cotton fields during the period of peak cotton susceptibility using visual and beat cloth methods [4,11,12]. Sampling accuracy for *L. lineolaris* may be impacted by the dispersal distance from the weedy hosts toward the interior of the cotton field [13]. Insects counts can be increased by the migrating insects, and higher adult counts were observed in cotton field plots with larger weed stand borders [13], potentially leading to additional insecticide applications. Monitoring the migratory movement of *L. lineolaris* between the wild host and the cotton crop is complex and is not well studied under the field conditions. Studies by Reisig [14] recommended sampling, using a sweep net, to be performed at locations not less than 15.3 m from the field edges to minimize the sample variation.

Insecticide applications based on well-informed scouting practices are critical for the management of this key pest. Prohibitory costs associated with the weekly scouting of the weedy field edges and adjacent cotton fields make it difficult for growers to make judicious decisions on insecticide applications. Monitoring tools based on visual cues and pheromones will be very useful in tracking *Lygus* populations in the wild hosts and weedy field edges early in the season to make informed decisions for pest management using insecticides. Previous studies have shown that the red-colored sticky cards are highly attractive to *L. lineolaris* adults compared to the blue, white, and yellow sticky cards. The red sticky cards baited with pheromone lures containing hexyl butyrate, (*E*)-2-hexenyl butyrate, and (*E*)-4-oxo-2-hexenal in a 4:10:7 ratio, respectively, trapped a significantly higher number of *L. lineolaris* than those lured with 10:4:2 or 7:10:4 blends or an unbaited control in the cotton field experiments [15].

The current study investigated the use of pheromone traps for monitoring *L. lineolaris* populations in different weed or wild hosts near cotton fields during the early spring season. The red and white sticky cards with or without pheromone lures were used. In three field experiments, we compared the effectiveness of the different traps for trapping *L. lineolaris* within a field of wild host plants, for monitoring the distribution and outward movement of *L. lineolaris* from a field of wild host plants during early spring before the crops were planted, and for monitoring the movement of *L. lineolaris* from wild hosts to cotton. Research findings from this study have field applications in using pheromone traps for the early season monitoring of *L. lineolaris* in the weedy field edges, which may contribute to effective weed and pest management strategies for row crops.

## 2. Materials and Methods

### 2.1. Study Locations and General Field Experiment Procedures

Field experiments were performed in two different field locations during the early spring and summer of 2022, in the Alcorn State University demonstration plot in Merigold, MS, and the USDA research farm, Southern Insect Management Research Unit, located in Stoneville, MS. For recording the catches of *L. lineolaris* on traps, the field-collected sticky cards were stored inside a Ziploc bag, and the number of *L. lineolaris* caught on the sticky cards was counted and recorded later in the laboratory. The male/female ratio of the insects was calculated for all the *L. lineolaris* trapped on the three out of the six replications of red and white sticky card color used. The species and sex of the captured *L. lineolaris* were determined based on the morphological characteristics of the abdomen. For female *L. lineolaris* adults, there is a groove that begins at the bottom and rises to the middle of her abdomen as the ovipositor lies in the center, almost hidden. This groove is not present for males and has a tapered abdomen [16,17,18]. The catches of *L. lineolaris* were analyzed via ANOVA followed by Tukey’s HSD to test for the significance of differences between means using the JMP statistical program (SAS, Cary, NC, USA).

### 2.2. Preparation of Lures and Traps

Pheromone lures used in the field experiments were prepared at the Natural Resources Institute, UK, according to George et al. [15]. Briefly, a blend of hexyl butyrate (HB), (*E*)-2-hexenyl butyrate (E2HB), and (*E*)-4-oxo-2-hexenal (E4OH) in a 40:100:70 ratio was formulated in sunflower oil with the major component at 10%. An antioxidant, 4-Methyl-2,6-di-*tert*-butylphenol (BHT; 10% of major component), and UV screener, Waxoline Black (10% of major component), were also added. The mixture (100 µL) was formulated on cigarette filters in pipette-tip dispensers (1 mL; Fisher Scientific, Loughborough, UK). The smaller end of the pipette tip was open and the larger end was sealed with a Teflon septum and crimp seal, before wrapping them with duct tape to exclude light. Aluminum foil bags were used to pack the lures and sent to USDA where they were stored in a refrigerator (4 °C) before use.

Red (Pherocon SWD trap; Trécé Inc., Adair, OK, USA) and non-UV white (Great Lakes IPM, Inc., Vestaburg, MI, USA) double-sided sticky cards (25 cm × 11.25 cm) were used in the experiments. The color and the hot melt glue on these sticky cards are effective in the monitoring and trapping of different insect species. The sticky cards were tied to a 30 cm × 2 mm-thick vinyl coated cable (Lowe’s Inc., Mooresville, NC, USA) and was attached to a 104 cm steel-painted metal traditional shepherd’s hook (LG Sourcing, Inc., North Wilkesboro, NC, USA) using gorilla black duct tape (Gorilla Glue Company, Cincinnati, OH, USA). Hanging the sticky cards to the shepherd’s hook helped to keep the sticky cards above the plant canopy and prevented contact with the plant. As these traps were deployed in the field for weeks under rainy and windy conditions, this setup allowed free movement, easy visibility, and prevented any wind damage to the sticky cards. The pheromone lures were attached horizontally to the center of the card using a plastic wire, with the pipette tip pointing away from the card [15].

### 2.3. Comparison of Different Colored Sticky Cards with Pheromone Lures for Monitoring Lygus lineolaris in Wild Hosts

The first experiment was performed at the Alcorn State University demonstration plot in early spring (7–21 April 2022) to study the attraction of *L. lineolaris* surviving on different weed or wild hosts towards the different colored double sticky cards with or without pheromone lures. A 0.30-hectare field plot with mustard (*Brassica juncea*) and other weed hosts were used for this experiment. Plants were in the flowering stage and the initial visual sampling showed a good population of *L. lineolaris*. Treatments included (1) red sticky card; (2) red sticky card + pheromone; (3) white sticky card; and (4) white sticky card + pheromone. Treatments were arranged 12 m apart in the same row and 5 m apart between rows. Traps were arranged in a completely randomized design and were replicated six times. Traps were arranged in a zig-zag pattern between the rows to allow for maximum spacing between the traps. The *L. lineolaris* that were trapped on the sticky cards were counted 7 days and 14 days after the deployment of the traps.

### 2.4. Perimeter Trapping of Lygus lineolaris Using Pheromone Traps

A second experiment was designed to study the dispersal and movement of *L. lineolaris* adults from wild hosts near a cotton and soybean field in early spring. The experiment field at the USDA research farm consisted of a 1-hectare rectangular plot with different weeds, wild grasses, and flowering plants. This plot was maintained as a pollinator plot throughout the year during the dearth of other pollen and honey resources in the offseason. During this experiment in early spring (20 April–18 May 2022), no other crops were planted next to this wildflower plot, and it provided wild hosts for feeding and survival early in the season. Treatments included (1) red sticky card; (2) red sticky card + pheromone; (3) white sticky card; and (4) white sticky card + pheromone. Treatments were set 15 m apart in the field borders covering all the four sides of this rectangular field. Traps were arranged randomly in a completely randomized design and were replicated six times. The numbers of *L. lineolaris* caught on the sticky cards were counted weekly for four weeks. The same pheromone lures were used throughout the duration of the experiment, and the sticky cards were replaced weekly.

### 2.5. Tracking Movement of Lygus lineolaris from Weed Hosts to Cotton Using Pheromone Traps

Multiple studies have reported that the overwintering *L. lineolaris* adults survive on weeds or other wild hosts in early spring before moving on to cotton and other row crops in the summer season. In a third experiment, the red and white sticky cards with or without pheromone lures were used to monitor the movement of *L. lineolaris* from the wildflowers to cotton. The pheromone traps covered all the four sides of the cotton field plot and were arranged to intercept the movement of the adults moving to the cotton crop from the wildflower plots. The experiment was performed at the Alcorn State University demonstration farm from 14 July–12 August 2022. Two patches of native wildflowers and other weeds (18.3 m wide, 0.41 hectare) were allowed to grow during the early spring season. These two wildflower beds were 30.5 m apart, and a plot of cotton (18.3 m wide, 0.41 hectare) was planted in early June in the middle of this space. A 6.1 m space was left clean without any weeds between the wildflowers and cotton on both sides of the cotton. Traps were arranged 15.3 m apart lengthwise in the center of this 6.1 m wide barrier space. The number of *L. lineolaris* caught on the sticky cards were counted weekly for four weeks. The same pheromone lures were used throughout the experiment, and the sticky cards were replaced weekly. Visual inspection of fruiting structures were randomly performed on forty plants (10 plants/row) in four rows of the experimental plots to check for the presence of *Lygus* adults and nymphs. The damage to the cotton squares was measured by counting the number of aborted squares on the plant. A total of 100 cotton squares was checked randomly and was replicated four times, and the percentage square retention was calculated.

## 3. Results

### 3.1. Comparison of Different Colored Sticky Cards with Pheromone Lures for Monitoring Lygus lineolaris in Weed Hosts

**Figure 1:**
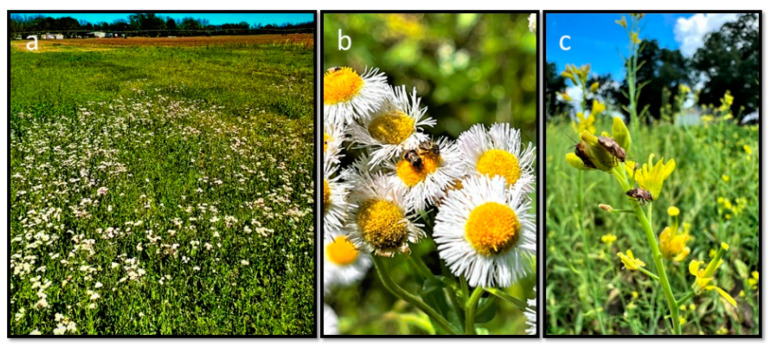
*Lygus lineolaris* adults on different types of wild hosts in early spring: (**a**) Patch of daisy fleabane (*Erigeron annus*) with *L. lineolaris* adults next to a cotton field in early spring; (**b**) *Lygus lineolaris* adults on daisy fleabane (*Erigeron annus*) flowers and (**c**) mustard (*Brassica juncea*) flowers in the early spring season.

**Figure 2:**
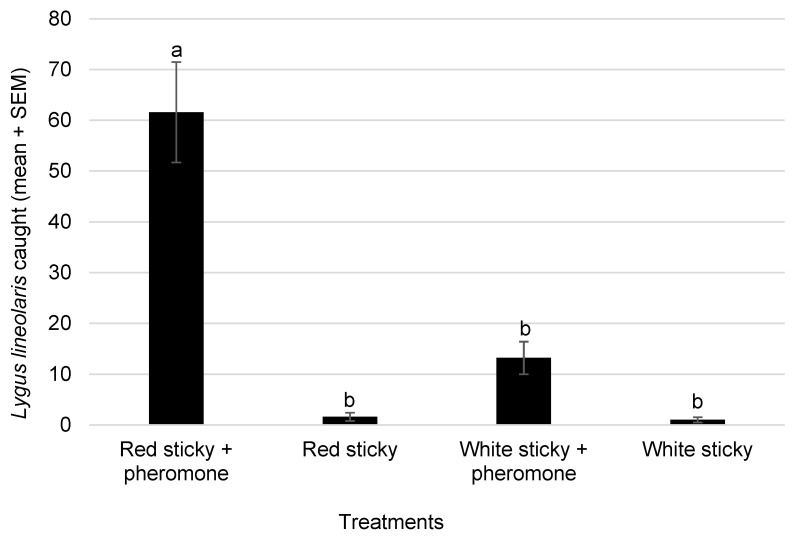
Mean (±SEM) cumulative catch of *Lygus lineolaris* caught on red and white sticky cards with or without pheromone lures in mustard, *Brassica junceae*, plot during early spring. The same pheromone lures were used during the two-week experiment (7–21 April 2022), and sticky cards were changed every week (*n* = 6; means with different letters are significantly different via Tukey’s HSD test after a significant ANOVA, *p* = 0.05).

The wild hosts contained a good number of *L. lineolaris* adults in early spring (Figure 1). The red sticky cards attached with pheromone lures caught significantly more adults than the white sticky cards with pheromone lures or the red and white sticky cards alone (*F*_3,23_ = 30.47; *p* < 0.001, *n* = 6) (Figure 2). After two weeks, the total *L. lineolaris* caught on the red sticky cards with pheromone lures were almost five times more than the white sticky cards with the same pheromone lure. A major portion of *L. lineolaris* collected on the sticky cards, irrespective of the treatments, were males, and the male/female percentage ratio was 97:3 for all the insects caught on the colored sticky cards. No differences were observed in the male/female ratio of *L. linoelaris* adults on the red or white sticky cards, with or without pheromones. The white sticky cards were observed to attract many other species of dipteran insects, compared to the red sticky cards with less bycatch of other insect species.

### 3.2. Perimeter Trapping of Lygus lineolaris Using Pheromone Traps

Monitoring the movement of *L. lineolaris* adults from the wildflowers was very effective using the sticky card traps with pheromone lures. The red sticky cards baited with pheromone lures caught the most number of *L. lineolaris* adults than the other sticky card treatments throughout the four weeks of the experiment (*p* < 0.005, *n* = 6) (Table 1).

**Figure 3:**
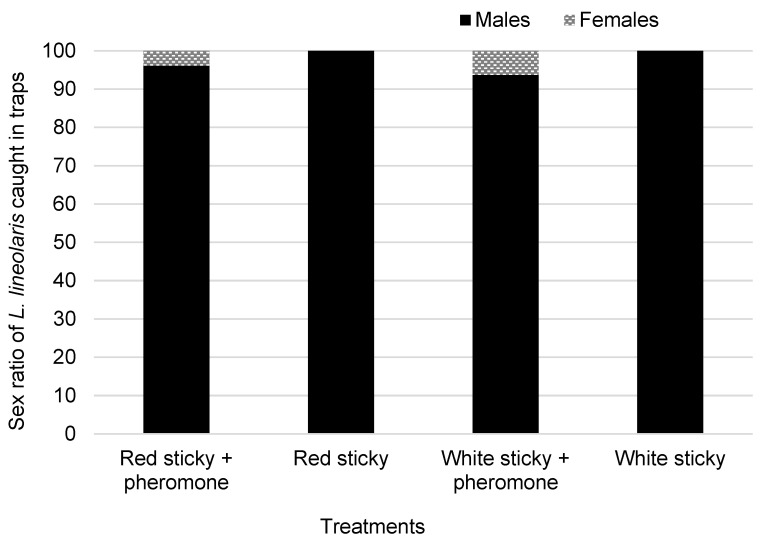
Male/female ratio of *Lygus lineolaris* caught on sticky card traps in the perimeter of weed hosts using sticky traps. Sex ratio is presented as the percentage of males/females (*n* = 152) caught on the traps. Experiments were performed in early spring (20 April–18 May 2022).

The total number of *L. lineolaris* caught on the red sticky card baited with pheromone was eight times higher than the white sticky cards with the same pheromone lure. The addition of the pheromone lure significantly increased the attraction towards the red sticky cards, although not towards the white sticky cards. The red and white sticky cards with or without the pheromone lure caught > 95% males compared to females (Figure 3). Few females were found on these traps irrespective of the color or presence of pheromone.

### 3.3. Tracking Lygus lineolaris Movement from Weed Hosts to Cotton Using Pheromone Traps

**Figure 4:**
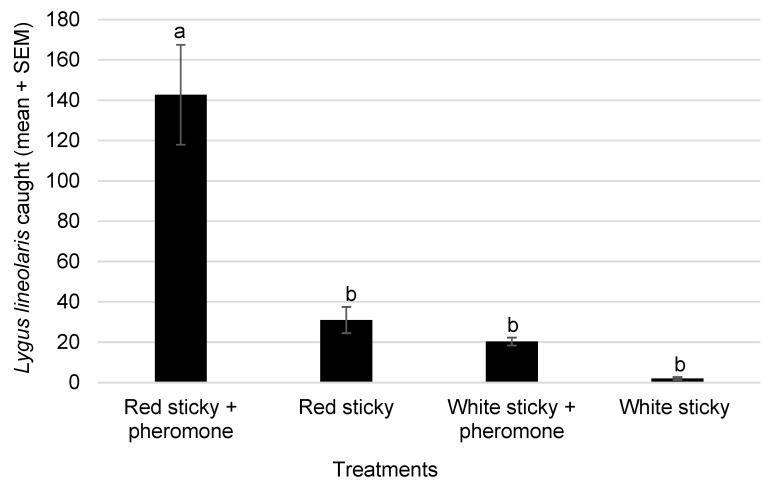
Mean (±SEM) cumulative number of adult *Lygus lineolaris* caught during five weeks (14 July–12 August 2022) on red or white sticky cards with or without pheromone lures placed in barrier rows between wild hosts and cotton (*n* = 6; means labeled with different letters are significantly different via Tukey’s HSD after a significant ANOVA; *p* = 0.05).

The red sticky cards caught significantly more *L. lineolaris* than the other trap treatments deployed in the experiment (*F*_3,23_ = 24.48; *p* < 0.0001, *n* = 6) (Figure 4). The sampling of the cotton plants and wild hosts visually during the second week of the experiment showed the presence of *L. lineolaris* adults in wild hosts (0.5 ± 0.2) and cotton (1.5 ± 0.6). At the end of the experiment, a square retention count was performed in cotton to assess the damage to the cotton squares. Cotton plants showed a 90% square retention even under the untreated conditions.

## 4. Discussion

*Lygus lineolaris* is a major hemipteran pest species in the Miridae family and causes extensive damage to agriculturally important crops. Like many other Mirids, their polyphagous feeding behavior, the wide host range, and their development of resistance to major insecticide classes makes *L. lineolaris* a very successful pest in cotton and other crops. The extensive application of malathion for boll weevil eradication has caused resistance development in *L. lineolaris* to organophosphates and pyrethroids in the mid-southern states [19,20]. Multiple studies have documented the presence of *L. lineolaris* on weed hosts near commercial crop field edges during the early spring season before they move on to commercial crop fields [7,10,13,21]. Scouting the susceptible stages of cotton using drop cloths, sweep nets, and visual observations on square retention and the number of dirty squares is important in making insecticide application decisions. However, there are limitations with these sampling methods depending on the insect and cotton growth stages [4,14,22]. The availability of alternate weed hosts in the field margins and the dispersal distance from these alternate hosts to the cotton interior may significantly affect these sampling techniques, which in turn may affect the management practices [13].

The detection and monitoring of pest species using pheromone traps have gained momentum in recent decades as it is ecofriendly, cost-effective, and contributes towards integrated pest management. Pheromone traps can be used for early season detection, season-long pest abundance monitoring, and mass trapping strategies. Pheromone traps that combine visual and pheromone cues may be very useful when monitoring *L. lineolaris* adults early in spring on different weedy hosts bordering the cotton fields. *Lygus lineolaris* pheromone components, their different blends, and their activity towards *Lygus* have been reported earlier [23,24,25,26,27]. Previous studies have field tested these lures using the white sticky cards against different *Lygus* spp. and have reported varying results in attracting *Lygus* adults [28,29]. George et al. [15] reported the use of pheromone lures to increase the attraction of *L. lineolaris* adults to the red colored sticky cards in cotton under the field conditions. Combining pheromone lures with the red colored sticky cards had a multiplicative effect on attracting and trapping *L. lineolaris* adults.

In the current study, a similar red sticky card pheromone combination was used to monitor the early season population of *L. lineolaris* in wild hosts, and their distribution and movement within and away from these alternate hosts. *Lygus* populations that multiply and build populations on weedy hosts need to be monitored and managed effectively before damage is sustained to the agriculturally important crops. Three set of experiments in two different locations investigated the efficacy of these pheromone traps in monitoring *L. lineolaris* movement in early spring. The first experiment showed that the red sticky cards baited with pheromone lure traps caught much higher numbers of *L. lineolaris* than the white sticky cards baited with pheromone or the sticky cards alone, and so are presumably more sensitive to detecting low populations. These pheromone traps were deployed in an area with different types of wild hosts, and the higher trap catches showed that these plants harbor a good number of *L. lineolaris* adults and act as breeding grounds before the agricultural crops are planted. The male/female ratio was 97:3, which is not surprising for an insect trap containing female-produced sex pheromone. However, the sticky cards without the pheromone lures also had > 90% males, which showed that there could be a higher proportion of males than females in the field, or the males are more active fliers, resulting in an increased number of males in our sticky cards.

A second experiment in wildflowers was also performed during the early spring season and our trapping results showed that there is a very active field population of *L. lineolaris*, and these adjacent weeds and wildflowers act as alternate hosts. These randomized treatment traps in the borders covering the whole 1-hectare plot trapped the insects that were moving in and out of this field. There was a higher proportion of males than females in the traps as in the previous experiment. If these insects are not managed before the cropping season, their movement into the neighboring crops can cause yield loss. Recent studies have shown that an early season infestation of *L. hesperus*, even at low densities, can result in significant yield loss, whereas a mid-season infestation of the same pest had only marginal effects on the cotton yield [30,31]. The distribution of weeds and alternate hosts in the field landscape can influence the movement of *L. lineolaris* [32,33]. Fleischer and Gaylor [21] previously reported the abundance of *L. lineolaris* on different types of weed hosts and how the weeds act as nurse crops for *L. lineolaris* early in the season. They have also reported that diversifying the agroecosystems may result in less damage to cotton, as multiple cultivated hosts will be available to feed on during the cropping season. Our experiments showed that the red sticky pheromone trap would be an effective tool for the early season monitoring of *L. lineolaris*.

The final experiment monitored the movement of *L. lineolaris* adults from wild hosts to the cotton crop during the summer season. The field sampling of wild hosts in the spring showed the presence of *L. lineolaris* in many of the wild hosts in the plots. Even though trapping *L. lineolaris* using sticky cards is not a preferred practice for pest management, the presence of these pheromone traps bordering the cotton plot may have contributed to a reduction in the movement of *L. lineolaris* from wild hosts to cotton. Very low populations of *L. lineolaris* were observed on the cotton plants and a 90% square retention rate was observed even without insecticide application. Using these pheromone traps and lure combinations will enable landscape-level sampling, reducing end-user variability, which continues to be a major problem in the scouting and monitoring of cotton insect pests. Effective weed management is important in reducing early *Lygus* populations that damage the developing fruiting structures, including the squares that critically impact the cotton yield.

Studies by D’Ambrosia et al. [13] described the development and seasonal movement of *L. lineolaris* from weeds into the cotton fields. The proximity to the weedy field edges can influence the dispersal of *L. lineolaris* late instar nymphs to the cotton crops. Mark-recapture studies have shown that *L. lineolaris* late instar nymphs can walk up to 50 m in situations where its weedy hosts were destroyed [34]. D’Ambrosio et al. [13] also reported that the flightless nymphs could reach cotton more readily from the declining weed hosts. Field weed edges with a higher perimeter relative to the area may also facilitate the movement of *L. lineolaris* into cotton as there is a higher weed–cotton interface. Flight mill studies have reported that adult *L. lineolaris* can travel 12 km/12 h [35], and 37.5 m/day in flowering cotton [36].

The current sampling strategies using drop cloths and sweep nets have advantages and disadvantages and require more human input and resources, in addition to the variability associated with these sampling methods. Monitoring pest populations using sticky cards and pheromone lures is cheap, effective, and requires less human input compared to the traditional sampling methods. These traps can be deployed early in the season to monitor the overwintering pest populations in weeds and other wild hosts. There are also options for automated pest monitoring, allowing growers to monitor pests caught on pheromone traps and send real-time trap catch information to computers or cell phones. These automatic traps are currently available and can be used for the continuous monitoring of pests based on which management decisions can be designed. The use of pheromone traps and automated trapping methods can help growers to optimize the use of pesticides and reduce insecticide residues. Combining the traditional sampling methods, pheromone traps, and automated trapping methods will help growers to monitor and make judicious decisions on managing the field weed edges that host early season pests.

## Figures and Tables

**Table 1:** Mean (±SEM) cumulative catch of *Lygus lineolaris* on red and white sticky cards with or without pheromone lures placed around the borders of a 1-hectare plot with wild hosts for five weeks during early spring. The same pheromone lures were used throughout the experiment and sticky cards were replaced every week (20 April–18 May 2022; *n* = 6); means followed by different letters within the same week are significantly different (*p* = 0.05).

Weeks after Deployment	Trap Color–Pheromone Treatments				*F*-Ratio	*p*-Value
	Red Sticky + Pheromone	Red Sticky	White Sticky + Pheromone	White Sticky		
Week 1	29.0 ± 4.1 ^a^	0.8 ± 0.4 ^b^	5.5 ± 0.8 ^b^	0.2 ± 0.2 ^b^	42.1	<0.001
Week 2	17.2 ± 3.6 ^a^	5.0 ± 2.7 ^b^	3.2 ± 0.9 ^b^	0.3 ± 0.3 ^b^	10.5	<0.001
Week 3	18.7 ± 4.4 ^a^	0.5 ± 0.2 ^b^	0.5 ± 0.3 ^b^	0 ± 0 ^b^	16.7	<0.001
Week 4	10.3 ± 3.7 ^a^	1.0 ± 0.6 ^b^	0.7 ± 0.3 ^b^	0.2 ± 0.2 ^b^	6.5	0.003

## Data Availability

All the data related to the research work are presented in the manuscript. Further details are available from the authors upon request.
